# Underweight in the First 2 Years of Life and Growth in Later Childhood

**DOI:** 10.1001/jamanetworkopen.2022.24417

**Published:** 2022-07-29

**Authors:** Courtney A. South, Charles D. G. Keown-Stoneman, Catherine S. Birken, Vasanti S. Malik, Stanley H. Zlotkin, Jonathon L. Maguire

**Affiliations:** 1 Department of Pediatrics, St Michael’s Hospital, Toronto, Canada; 2 Department of Nutritional Sciences, Faculty of Medicine, University of Toronto, Toronto, Canada; 3 Li Ka Shing Knowledge Institute, St Michael’s Hospital, Toronto, Canada; 4 Biostatistics Division, Dalla Lana School of Public Health, University of Toronto, Toronto, Canada; 5 Child Health Evaluative Sciences, Hospital for Sick Children, Toronto, Canada; 6 Institute of Health Policy, Management and Evaluation, Dalla Lana School of Public Health, University of Toronto, Toronto, Canada; 7 Department of Paediatrics, Faculty of Medicine, University of Toronto, Toronto, Canada; 8 Department of Nutrition, Harvard T.H. Chan School of Public Health, Boston, Massachusetts; 9 Centre for Global Child Health, Hospital for Sick Children, Toronto, Canada; 10 Epidemiology Division, Dalla Lana School of Public Health, University of Toronto, Toronto, Canada

## Abstract

**Importance:**

Few studies have examined the association between underweight in the first 2 years and growth in later childhood in high-income countries.

**Objective:**

To evaluate the associations of underweight in the first 2 years of life with body mass index (calculated as weight in kilograms divided by height in meters squared) *z* score (zBMI), weight-for-age *z* score (WAZ), and height-for-age *z* score (HAZ) from ages 2 to 10 years.

**Design, Setting, and Participants:**

This prospective cohort study was conducted between February 2008 to September 2020 in The Applied Research Group for Kids! practice-based research network in Toronto, Canada. Participants included healthy children aged 0 to 10 years. Data were analyzed from October 2020 to December 2021.

**Exposures:**

Underweight (ie, zBMI less than −2, per the World Health Organization) in the first 2 years of life.

**Main Outcomes and Measures:**

The primary outcome was zBMI from ages 2 to 10 years. Linear mixed-effects models were used to account for multiple growth measures over time.

**Results:**

A total of 5803 children were included in the primary analysis. At baseline, the mean (SD) age was 4.07 (5.62) months, 2982 (52.2%) were boys, and 550 children (9.5%) were underweight. Underweight in the first 2 years was associated with lower zBMI (difference, −0.39 [95% CI, −0.48 to −0.31]) at 10 years and lower HAZ (difference, −0.24 [95% CI, −0.34 to −0.14]) at age 2 years. Stratified by sex, at age 10 years, girls and boys with underweight in the first 2 years both had lower zBMI (girls: difference, −0.47 [95% CI, −0.59 to −0.34]; boys: difference, −0.32 [95% CI, −0.44 to −0.20]). At age 10 years, children with underweight and a lower zBMI growth rate in the first 2 years had lower zBMI (difference, −0.64 [95% CI, −0.77 to −0.53) and HAZ (difference, −0.12 [−0.24 to −0.01]), while children with underweight and a higher zBMI growth rate in the first 2 years had similar zBMI (difference, −0.11 [95% CI, −0.22 to 0.001]) and higher HAZ (difference, 0.16 [95% CI, 0.05 to 0.27]) compared with children who did not have underweight in the first 2 years.

**Conclusions and Relevance:**

In this prospective cohort study, children with underweight in the first 2 years of life had lower zBMI and HAZ in later childhood. These associations were attenuated among children with a higher growth rate in the first 2 years.

## Key Points

**Question:**

Is underweight in the first 2 years of life associated with lower growth in later childhood?

**Findings:**

This cohort study of 5803 children found that children with underweight in the first 2 years had lower body mass index *z* score at age 10 years. These difference were greater for girls and children with lower growth rate in the first 2 years.

**Meaning:**

This cohort study found that underweight in the first 2 years was associated with lower body mass index *z* score through age 10 years.

## Teaser

This cohort study assesses the associations of underweight in the first 2 years of life with body mass index, weight-for-height, and height-for-age *z* scores at ages 2 to 10 years.

## Introduction

The first 2 years of life are important for establishing health over the life course.^1,2^ Growth is highest in the first 2 years compared with any other life stage.^3,4^ Underweight in children has been defined as body mass index (calculated as weight in kilograms divided by height in meters squared) *z* score (zBMI) less than −2 by the World Health Organization (WHO),^5,6,7^ and it may occur when energy expenditure is greater than intake.^8,9,10,11^ In a healthy population, given biological variation, approximately 2% of children’s zBMI would be expected to be less than −2.^5^ The prevalence of underweight in the first 2 years in high-income countries has been reported to be approximately 7%, which is higher than both overweight (5.6%), and obesity (1%).^12,13,14^ Growth monitoring is recommended as an indicator of adequate nutrition.^15,16,17^ A child’s growth rate is determined by tracking multiple growth measurements over time and comparing them to growth standards for age and sex.^5,16,18,19^ Growth standards from the WHO are recommended for use by health care practitioners to determine if a child is growing as expected and identify children with a lower or higher growth rate.^5,6^

The reasons why young children may have underweight in high-income countries are believed to be multifactorial, including insufficient income to afford food, feeding problems, and poor eating behaviors.^20,21,22,23,24^ Few long-term studies in high-income countries have evaluated underweight in the first 2 years and growth in later childhood.^20,25,26,27^ A 2005 systematic review by Rudolf et al^25^ that included 3 small case-control studies, 2 cohort studies, and 2 randomized clinical trials from high-income countries, with sample sizes between 13 and 229 children, found that children younger than 2 years with weight or rate of weight gain in lower than the 10th percentile had lower weight and height at ages 3 to 9 years. In meta-analysis, Rudolf et al^25^ pooled data from 2 studies that included 90 children with weight or rate of weight gain in lower than the 10th percentile and found that weight was lower by 1.2 (95% CI, −0.5 to −2.0) SD and height was lower by 0.9 (95% CI, −0.3 to −1.5) SD at age 6 years. Rudolf et al^25^ questioned the clinical importance of these findings, owing to relatively short follow up, small sample sizes, and inadequate adjustment for confounders. Lack of evidence has led to inconsistent recommendations by health care practitioners.^20,25,28^

The primary objective of this study was to evaluate the association between underweight in the first 2 years and zBMI in later childhood. Secondary objectives included examining weight-for-age *z* score (WAZ) and height-for-age *z* score (HAZ) in later childhood, as well as exploring whether sex and zBMI growth rate in the first 2 years modified these associations. We hypothesized that children with underweight in the first 2 years would have lower zBMI, lower WAZ, and lower HAZ in later childhood and the differences would be most pronounced for children with underweight and lower growth rate in the first 2 years.

## Methods

This cohort study was approved by the Research Ethics Board at the Hospital for Sick Children and St Michael’s Hospital, and all parents of participating children provided written informed consent. This study followed Strengthening the Reporting of Observational Studies in Epidemiology (STROBE) reporting guideline for observational cohort studies.

This was a prospective cohort study conducted through The Applied Research Group for Kids! (TARGet Kids!) practice-based research network in Canada.^29^ Children aged 2 years and younger were recruited between February 2008 and September 2020 during well-child visits at TARGet Kids! participating clinics. Healthy children were included if they had at least 1 zBMI measurement from ages 0 to 2 years and at least 1 zBMI measurement between ages 2 to 10 years. Children were excluded if they had a health condition affecting growth or were born premature (<37 weeks gestation) at enrollment.

Data were collected from parents using a standardized questionnaire adapted from the Canadian Community Health Survey.^29^ Trained research assistants or health care practitioners obtained weight in kilograms and height in centimeters at each well-child visit using a standardized approach.^30^ Weight was measured using a precision digital scale.^30^ Height was measured with a length board for children younger than age 2 years and with a calibrated stadiometer for children older than age 2 years.^30^ zBMI, WAZ, and HAZ were age- and sex-standardized using WHO growth standards.^6,31^

The primary exposure variable was the first underweight measurement, which occurred at any time point between age 0 and 2 years and was defined as zBMI less than −2, per the WHO.^5,6^ The primary outcome was zBMI between ages 2 and 10 years. Secondary outcomes were WAZ and HAZ using WHO growth standards at the same ages.^6^ The WHO cutoffs were used to examine weight status categorically, which were defined as underweight, less than −2; normal weight, −2 to 1; overweight, 1 to 2; and obese, greater than 2. Low WAZ and HAZ were each defined as less than −2.^31,32^

Potential confounders were identified a priori from the review of the literature. These included birthweight,^33,34,35^ breastfeeding duration, parent BMI, parent height, maternal ethnicity, and parent-reported family income.^10,35^ Parent anthropometrics were obtained by research assistants at each well-child visit.^29^ Breastfeeding duration was measured by the question ”Has your child ever been breastfed?” If the child was currently breastfeeding, the child’s age at that time was used or the parent indicated when breastfeeding was discontinued to determine the duration of breastfeeding. Maternal ethnicity and family income were categorized and self-reported by parents.

### Statistical Analysis

Descriptive analysis was completed to determine the mean (SD) or proportion (%) for baseline participant characteristics. Linear mixed-effects models (LMM) were used to evaluate the primary and secondary analysis, adjusting for the potential confounding variables.^36^ LMM allowed for multiple growth measurements over time to be evaluated at the individual level, using random effects to account for correlation among measurements from the same individual and fixed effects for other covariates in the model.^36^ Multinomial generalized estimating equations were used to estimate the associations of the exposure with WHO-defined weight status categories. Logistic generalized LMM were used to estimate the association of the exposure with low WAZ, and low HAZ. Owing to the previously observed nonlinear associations between *z* scores and age, restricted cubic splines fitted with 5 knots at prespecified percentile location of the age distribution in the continuous outcome period were used.^37^ From the fitted models, growth over time was estimated and age was measured continuously, with contrasts provided at ages 2, 5, and 10 years. To explore potential modification, an interaction term for sex was included in each model and assessed using a likelihood ratio test.

For the secondary analysis, LMMs were used to explore how zBMI growth rate in the first 2 years of life modified the association between underweight in the first 2 years and growth from ages 2 to 10 years. At least 2 zBMI measurements from ages 0 to 2 years were required for children with underweight in the secondary analysis. LMM used repeated growth measures to estimate individual zBMI growth rates from birth to age 2 years as the participant-specific slope. The zBMI growth rates were standardized to a mean of 0.^38^ Underweight and lower zBMI growth rate in the first 2 years was defined as 1 SD less than the mean, and underweight and higher zBMI growth rate in the first 2 years was defined as 1 SD greater than the mean.^37^ To evaluate association modification, zBMI growth rate, as measured by the participant-specific slope was included as an interaction term in each model and assessed using a likelihood ratio test.

Missing data for the covariates, exposure, and outcomes were all less than 17%. All missing data were assumed to be missing at random, conditional on the other variables in the model. Multiple imputation using 20 data sets was used for covariates, exposure, and outcome variables to reduce the bias of missing data.^39^ Residuals and Q-Q plots were assessed to determine the adequacy of model fit. Results were considered statistically significant with *P* = .05. LMM, generalized estimating equations, and generalized LMM were 2-sided, and likelihood ratio tests were 1-sided. All statistical analyses were completed using R statistical software version 4.0.3 (R Project for Statistical Computing). Data were analyzed from October 2020 to December 2021.

## Results

**Figure 1.**
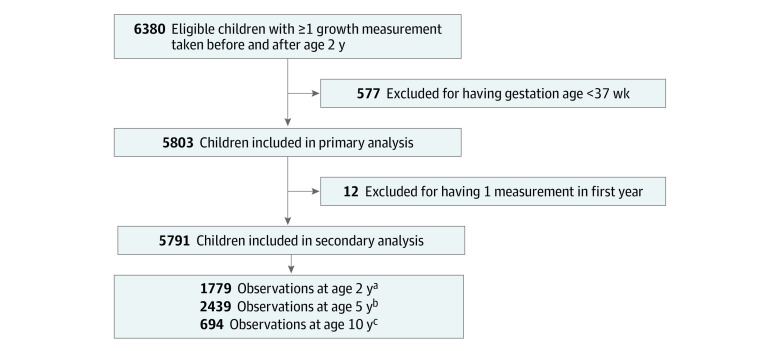
Flowchart of Participants Recruited and Included for Analysis Implausible growth measurements were flagged if height-for-age was less than −6.0 and more than 6.0; weight-for-age was less than −6.0 and more than 5.0; and body mass index (calculated as weight in kilograms divided by height in meters squared) *z* score was less than −5.0 and more than 5.0. Parent body mass index was also flagged if deemed implausible for mother (<18.8 and >48.0), and father (<19.4, >42.7). Measurements were set to missing if the growth measurement from the prior or following visit was more than 2 SD above or below the outlier or if the growth measurement on the original data collection form was also an outlier. There were 368 implausible observations of child body mass index, height-for-age, weight for age, parent body mass index *z* scores. ^a^Age 2 years is defined as children who attended a visit between ages 25 and 34 months. ^b^Age 5 years is defined as children who attended a visit between ages 57 and 68 months. ^c^Age 10 years is defined as children who attended a visit between ages 117 and 128 months.

A total of 5803 children (mean [SD] age at baseline 4.07 (5.62); 2982 [52.2%] boys) were included (Figure 1). Table 1 shows the summary of child characteristics at enrollment. Self-reported maternal ethnicity included 282 African participants (5.5%); 908 East, South, or Southeast Asian participants (17.7%); 3357 European participants (65.6%); and 568 participants (9.8%) who reported more than 1 ethnicity or Latin American, Arab, North American Aboriginal, or Oceanian ethnicity. At baseline, there were 550 children (9.5%) with underweight in the first 2 years of life, and children with underweight were more likely to be younger, have lower birthweight, and more likely to report Asian maternal ethnicity (Table 1). In the primary analysis, children with underweight in the first 2 years had lower zBMI at ages 2 years (difference, −0.58 [95% CI, −0.69 to −0.48]), 5 years (difference, −0.49 [95% CI, −0.56 to −0.41]), and 10 years (difference, −0.39 [95% CI, −0.48 to −0.31) compared with children who did not have underweight in the first 2 years (Table 2). For example, it was estimated that children with underweight in the first 2 years had a mean of 1.23 (95% CI, −1.51 to −0.98) kg lower weight at age 10 years.

**Table 1.** Baseline Child and Family Characteristics Among Participants With and Without Underweight in the First Two Years of Life^a^

Variable	Children, mean (SD)		
	Not underweight (n = 5159)^b^	Underweight (n = 550)^c^	All (N = 5803)
Age, mo	4.20 (5.73)	2.41 (3.66)	4.07 (5.62)
Birthweight, kg	3.41 (0.51)	2.97 (0.59)	3.37 (0.53)
Sex, No. (%)			
Boys	2671 (52.2)	246 (51.1)	2982 (52.2)
Girls	2449 (47.8)	246 (48.9)	2730 (47.8)
zBMI	−0.32 (0.98)	−2.66 (0.61)	−0.55 (1.17)
WAZ	0.05 (0.98)	−1.84 (1.27)	0.44 (1.43)
HAZ	0.50 (1.32)	−0.03 (2.13)	−0.13 (1.16)
Breastfeeding duration, mo	9.12 (6.58)	8.33 (5.99)	9.03 (6.53)
Parent height, cm^d^	166.0 (8.59)	165.3 (8.98)	165.9 (8.65)
Parent BMI			
Both^d^	25.36 (5.01)	25.02 (4.79)	25.34 (5.02)
Mother’s measurement used, No. (%)^e^	4253 (84.2)	406 (82.2)	4725 (83.9)
Maternal ethnicity, No. (%)^f^			
African	243 (5.3)	36 (8.2)	282 (5.5)
East, South, or Southeast Asian	790 (15.3)	106 (19.2)	908 (17.7)
European	3046 (66.3)	253 (57.6)	3357 (65.6)
>1 Ethnicity, Latin American, Arab, North American Aboriginal, and Oceanian	516 (10.0)	44 (8.0)	568 (9.8)
Self-reported family income, CAD$, No. (%)^g^			
$0-39 999	400 (7.8)	49 (8.9)	456 (7.85)
$40 000-$79 999	585 (11.3)	60 (10.9)	659 (11.3)
$80 000-$149 999	1425 (27.6)	131 (23.8)	1578 (27.2)
≥$150 000	1917 (37.2)	177 (32.2)	2133 (36.7)

Abbreviations: HAZ, height-for-age *z* score; zBMI, body mass index (calculated as weight in kilograms divided by height in meters squared) *z* score; WAZ, weight-for-age *z* score.

^a^ Data were collected at enrollment, and numbers may not add up to total owing to missing values.

^b^ Not underweight was defined as zBMI of −2 or greater, per the World Health Organization age and sex standardization.

^c^ Underweight was defined as zBMI less than −2, per the World Health Organization age and sex standardization.

^d^ Both maternal and paternal measurements used, value represents the mean of all parent measurements.

^e^ Represents the number and percentage of mothers who were measured for parent BMI and parent height.

^f^ Maternal ethnicity was defined as the self-reported ethnicity of the child’s biological mother.

^g^ In US$, self-reported family income quartiles were $0 to $30 926.43, $30 927.20 to $61 853.63, $61 854.40 to $115 976.23, and $115 977.00 or more.

**Table 2.** Estimated Differences and Odd Ratios in Growth, for Underweight and Not Underweight in the First 2 Years of Life for All Children and Modified by Sex

Age, y^a^	Estimate (95% CI)			Odds ratio (95% CI)					
	zBMI^b^	WAZ^b^	HAZ^b^	Underweight^b^^,^^c^	Overweight^b^^,^^c^	Obese^b^^,^^c^	Low WAZ^b^^,^^d^	Low HAZ^b^^,^^e^
**All**										
2	−0.58 (−0.69 to −0.48)	−0.54 (−0.63 to −0.45)	−0.24 (−0.34 to −0.14)	7.51 (4.02 to 14.03)	0.39 (0.30 to 0.50)	0.33 (0.20 to 0.57)	5.05 (2.71 to 9.39)	1.58 (1.07 to 2.32)
5	−0.49 (−0.56 to −0.41)	−0.37 (−0.43 to −0.30)	−0.07 (−0.15 to 0.01)	3.26 (2.15 to 4.93)	0.54 (0.44 to 0.66)	0.55 (0.39 to 0.78)	4.11 (2.89 to 5.86)	1.49 (1.09 to 2.0)
10	−0.39 (−0.48 to −0.31)	−0.26 (−0.34 to −0.18)	0.01 (−0.07 to 0.10)	3.62 (2.11 to 6.23)	0.66 (0.46 to 0.93)	0.78 (0.53 to 1.17)	1.99 (1.27 to 3.09)	0.99 (0.59 to 1.6)
**Boys**										
2	−0.59 (−0.73 to −0.45)	−0.49 (−0.61 to −0.37)	−0.15 (−0.29 to −0.02)	18.83 (2.66 to 133.05)	0.41 (0.18 to 0.94)	0.65 (0.12 to 3.42)	4.13 (1.35 to 12.64)	1.19 (0.59 to 2.43)
5	−0.50 (−0.60 to −0.40)	−0.34 (−0.44 to −0.25)	−0.03 (−0.13 to 0.07)	10.13 (0.76 to 134.23)	0.57 (0.19 to 1.67)	1.25 (0.16 to 10.01)	3.66 (1.92 to 6.96)	1.36 (0.77 to 2.40)
10	−0.32 (−0.44 to −0.20)	−0.19 (−0.30 to −0.08)	0.05 (−0.06 to 0.17)	7.99 (0.64 to 99.34)	0.85 (0.30 to 2.41)	1.88 (0.23 to 15.39)	1.32 (0.51 to 3.41)	0.45 (0.10 to 2.08)
**Girls**										
2	−0.56 (−0.71 to −0.41)	−0.59 (−0.72 to −0.46)	−0.33 (−0.48 to −0.18)	10.77 (4.31 to 26.93)	0.41 (0.28 to 0.59)	0.43 (0.19 to 0.95)	6.07 (1.84 to 20.09)	2.04 (0.98 to 4.23)
5	−0.47 (−0.58 to −0.37)	−0.39 (−0.49 to −0.29)	−0.11 (−0.22 to −0.01)	3.15 (1.76 to 5.64)	0.53 (0.39 to 0.73)	0.49 (0.28 to 0.88)	4.80 (2.48 to 9.27)	1.74 (0.98 to 3.06)
10	−0.47 (−0.59 to −0.34)	−0.34 (−0.45 to −0.22)	−0.03 (−0.16 to 0.09)	6.14 (2.79 to 13.52)	0.53 (0.31 to 0.91)	0.59 (0.31 to 1.15)	2.63 (1.23 to 5.62)	1.29 (0.59 to 2.84)

Abbreviations: HAZ, height-for-age *z* score; zBMI, body mass index (calculated as weight in kilograms divided by height in meters squared) *z* score; WAZ, weight-for-age *z* score.

^a^ Age in years at the time of outcome measurement of growth.

^b^ Adjusted for breastfeeding duration, child sex (for overall analysis), birthweight, parent BMI, parent height, maternal ethnicity, and family income.

^c^ Normal weight was defined as zBMI −2 to 1; underweight, zBMI less than −2; overweight, zBMI greater than 1 to 2; and obese, zBMI greater than 2.

^d^ Low weight-for-age defined as WAZ less than −2.

^e^ Low height-for-age defined as HAZ less than −2.

Underweight in the first 2 years was associated with lower HAZ at age 2 years (difference, −0.24 [95% CI, −0.34 to −0.14]), but that difference was attenuated at ages 5 years (difference, −0.07 [95% CI, −0.15 to 0.01) and 10 years (difference, 0.01 [95% CI, −0.07 to 0.10). For example, children with underweight in the first 2 years were a mean of 0.68 (95% CI, −1.05 to −0.43) cm shorter at age 2 years, compared with children who did not have underweight.

**Figure 2.**
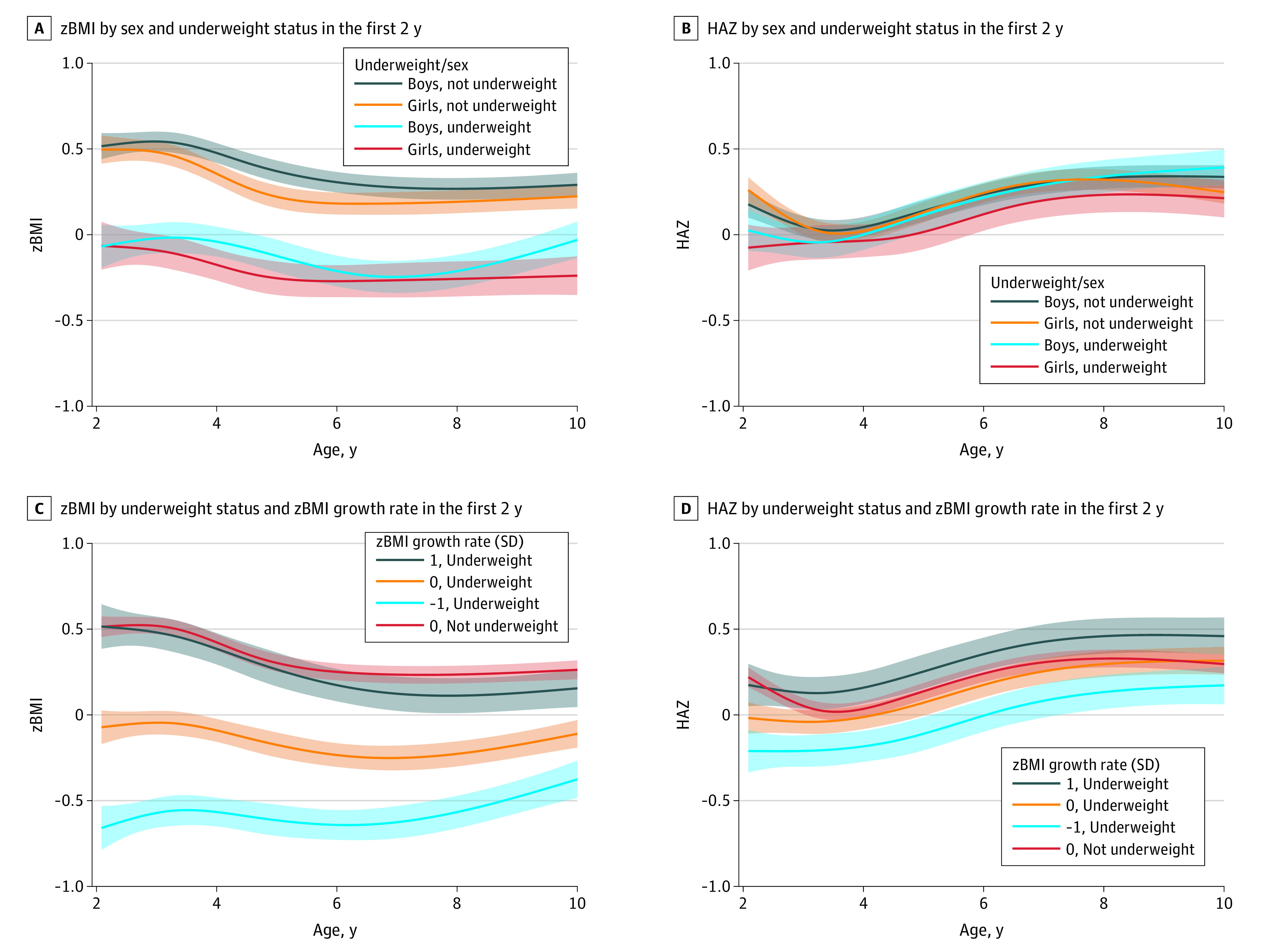
Body Mass Index *z* Score (zBMI) and Height-for-Age *z* Score (HAZ) Growth From Ages 2 to 10 Years by Underweight Status, Sex, and the zBMI Growth Rate in the First 2 Years of Life Estimates are adjusted for breastfeeding duration, birthweight, parent BMI (calculated as weight in kilograms divided by height in meters squared), parent height, maternal ethnicity, and family income. C and D are additionally adjusted for sex.

Sex was associated with modifying the association between underweight in the first 2 years and zBMI (χ^2^ = 2.70; *P* = .004), WAZ (χ^2^ = 3.73; *P* < .001), and HAZ (χ^2^ = 12.68; *P* < .001) in later childhood, evaluated through likelihood ratio tests. Figure 2 shows zBMI and HAZ growth from ages 2 to 10 years by sex and underweight status in the first 2 years. Girls with underweight in the first 2 years had lower zBMI (difference, −0.47 [95% CI, −0.59 to −0.34]) and higher odds of underweight (odds ratio [OR], 6.14 [95% CI, 2.79 to 13.52]) and were a mean of 1.63 (95% CI, −2.04 to −1.28) kg lighter at age 10 years compared with girls without underweight (Table 2). Boys with underweight in the first 2 years had lower zBMI (difference, −0.32 [95% CI, −0.44 to −0.20]) and were a mean of 0.91 (95% CI, −1.25 to −0.57) kg lighter at age 10 years compared with boys without underweight. Girls with underweight in the first 2 years had lower HAZ at ages 2 years (difference, −0.33 [95% CI, −0.48 to −0.18]) and 5 years (difference, −0.11 [95% CI, −0.22 to −0.01]). For example, girls with underweight in the first 2 years were a mean of 1.05 (95% CI, −1.54 to −0.58) cm shorter at age 2 years and 0.54 (95% CI, −0.86 to −0.04) cm shorter at age 5 years, but there was no significant difference at age 10 years (difference, −0.03 [95% CI, −0.16 to 0.09]). Boys with underweight in the first 2 years had lower HAZ (difference, −0.15 [95% CI, −0.29 to −0.02]) and were a mean of 0.45 (95% CI, −0.87 to −0.06) cm shorter at age 2 years, but there was no significant difference at ages 5 years (difference, −0.03 [95% CI, −0.13 to 0.07]) or 10 years (difference, 0.05 [95% CI, −0.06 to 0.17).

The growth rate of underweight children in the first 2 years modified the association with zBMI (χ^2^ = 80.23; *P* < .001), WAZ (χ^2^ = 65.32; *P* < .001), and HAZ (χ^2^ = 23.05; *P* < .001) in later childhood, evaluated through likelihood ratio tests. Figure 2 shows zBMI and HAZ from ages 2 to 10 years by underweight status and zBMI growth rate in the first 2 years. Children with underweight and 1 SD lower growth rate in the first 2 years had lower zBMI, WAZ, and HAZ through age 10 years (Table 3). At age 10 years, children with underweight and lower zBMI growth rate in the first 2 years had lower zBMI (difference, −0.64 [95% CI, −0.77 to −0.53]), higher odds of underweight (OR, 5.60 [95% CI, 3.15 to 9.96), and lower HAZ (difference, −0.12 [95% CI, −0.24 to −0.01]) compared with children without underweight. For example, at age 10 years, children with underweight and lower zBMI growth rate in the first 2 years were a mean of 2.02 (95% CI, −2.43 to 1.67) kg lighter and 0.79 (95% CI, −1.54 to −0.06) cm shorter.

**Table 3.** Estimated Differences and Odd Ratios in Growth for Underweight and Not Underweight in the First 2 Years of Life, Modified by zBMI Growth Rate

zBMI rate (SD)	Estimate (95%CI)^a^			Odds ratio (95%CI)^a^^,^^b^				
	zBMI	WAZ	HAZ	Underweight	Overweight	Obese	Low WAZ^c^	Low HAZ^d^
**Age 2 y^e^**								
Low^f^	−1.17 (−1.31 to −1.04)	−1.06 (−1.18 to −0.94)	−0.43 (−0.56 to −0.30)	11.75 (6.21 to 22.22)	0.09 (0.05 to 0.16)	0.03 (0.01 to 0.11)	10.27 (5.43 to 19.41)	2.19 (1.37 to 3.49)
Average^g^	−0.59 (−0.69 to −0.48)	−0.55 (−0.64 to −0.45)	−0.24 (−0.34 to −0.14)	4.82 (2.36 to 9.79)	0.26 (0.19 to 0.36)	0.16 (0.08 to 0.34)	3.15 (1.60 to 6.22)	1.56 (1.06 to 2.30)
High^h^	<0.01 (−0.13 to 0.13)	−0.03 (−0.15 to 0.09)	−0.04 (−0.17 to 0.09)	1.97 (0.76 to 5.14)	0.76 (0.57 to 1.01)	0.79 (0.48 to 1.31)	0.97 (0.39 to 2.39)	1.11 (0.67 to 1.84)
**Age 5 y^a^**								
Low^f^	−0.92 (−1.01 to −0.82)	−0.76 (−0.84 to −0.67)	−0.25 (−0.35 to −0.15)	5.38 (3.52 to 8.22)	0.13 (0.09 to 0.19)	0.19 (0.01 to 0.36)	6.56 (4.54 to 9.48)	1.53 (1.03 to 2.26)
Average^g^	−0.48 (−0.55 to −0.40)	−0.36 (−0.43 to −0.29)	−0.07 (−0.14 to 0.01)	1.92 (1.2 to 3.07)	0.38 (0.30 to 0.48)	0.43 (0.29 to 0.60)	3.00 (2.05 to 4.39)	1.47 (1.08 to 2.00)
High^h^	−0.04 (−0.14 to 0.06)	0.04 (−0.05 to 0.13)	0.12 (0.02 to 0.22)	0.68 (0.35 to 1.34)	1.08 (0.86 to 1.36)	0.99 (0.67 to 1.45)	1.37 (0.82 to 2.26)	1.42 (0.96 to 2.10)
**Age 10 y^a^**								
Low^f^	−0.64 (−0.77 to −0.53)	−0.51 (−0.61 to −0.41)	−0.12 (−0.24 to −0.01)	5.60 (3.15 to 9.96)	0.75 (0.49 to 1.1)	0.48 (0.21 to 1.12)	2.04 (1.45 to 2.88)	0.99 (0.46 to 2.14)
Average^g^	−0.38 (−0.46 to −0.29)	−0.25 (−0.32 to −0.17)	0.02 (−0.07 to 0.10)	2.71 (1.51 to 4.86)	0.65 (0.47 to 0.90)	0.74 (0.46 to 1.19)	1.70 (1.06 to 2.72)	0.99 (0.58 to 1.70)
High^h^	−0.11 (−0.22 to <0.01)	0.02 (−0.08 to 0.12)	0.16 (0.05 to 0.27)	1.31 (0.60 to 2.85)	0.55 (0.35 to 0.88)	1.15 (0.75 to 1.75)	0.83 (0.43 to 1.60)	0.99 (0.51 to 1.90)

Abbreviations: HAZ, height-for-age *z* score; zBMI, body mass index (calculated as weight in kilograms divided by height in meters squared) z score; WAZ, weight-for-age *z* score.

^a^ Adjusted for breastfeeding duration, child sex, birthweight, parent BMI, parent height, maternal ethnicity, and family income.

^b^ Normal weight was defined as zBMI −2 to 1; underweight, zBMI less than −2; overweight, zBMI greater than 1 to 2; obese, zBMI greater than 2.

^c^ Low weight-for-age defined as WAZ less than −2.

^d^ Low height-for-age defined as HAZ less than −2.

^e^ Age in years at the time of outcome measurement of growth.

^f^ Lower zBMI growth rate are children with underweight with 1 SD unit lower zBMI growth rate than the mean (0 SD) in the first 2 years of life.

^g^ Average zBMI growth rate are children with underweight with 0 SD unit zBMI growth rate in the first 2 years of life.

^h^ Higher zBMI growth rate are children with underweight with 1 SD unit higher zBMI growth rate than the mean (0 SD) in the first 2 years of life.

Children with underweight and 1 SD higher growth rate in the first 2 years had similar mean zBMI (difference, −0.11 [95% CI, −0.22 to 0.001]) and lower odds of overweight (OR, 0.55 [95% CI, 0.35 to 0.88]) at age 10 years. They had higher HAZ at age 10 years (difference, 0.16 [95% CI, 0.05 to 0.27). For example, at age 10 years, children with underweight and a higher zBMI growth rate in the first 2 years were a mean of 1.06 (95% CI, 0.32 to 1.73) cm taller than children without underweight.

## Discussion

In this prospective cohort study, healthy urban children with underweight in the first 2 years of life had lower mean zBMI and similar mean HAZ at age 10 years compared with children without underweight. At age 10 years, girls with underweight in the first 2 years had a mean of 1.6 kg lower weight and boys with underweight had a mean of 0.9 kg lower weight. Children with underweight and a lower growth rate in the first 2 years were a mean of 2.0 kg lighter and 0.8 cm shorter at age 10 years compared with children without underweight. Children with underweight and a higher growth rate in the first 2 years had similar zBMI and higher HAZ at age 10 years. At age 10 years, female sex was associated with 6.1-fold higher odds of underweight, and a lower growth rate in the first 2 years was associated with 5.6-fold higher odds of underweight, suggesting that underweight in the first 2 years of life may have clinically meaningful growth outcomes in later childhood. Taken together, these results suggest that underweight in the first 2 years of life, particularly among girls and children with a lower growth rate, was associated with lower zBMI at age 10 years, and lower growth rate was associated with lower HAZ at age 10 years.

The WHO, the American Academy of Pediatrics, and the Royal College of Paediatrics and Child Health provide guidelines on the prevention and treatment of obesity in young children but not for underweight in high-income countries.^40,41,42^ Results from this study highlight that further research is needed to improve recommendations for children with underweight, especially for girls and those with a lower growth rate. For example, health care practitioners may reinforce best practices for breastmilk or cow’s milk intake, promote an energy-dense diet, improve eating behaviors and skills, and recommend childcare attendance, as well as address socioeconomic needs, such as insufficient income to afford food.^8,13,20^

Findings from this study complement and extend findings from other studies that have investigated underweight in high-income countries. In the United Kingdom, Wright et al^43^ conducted a prospective cohort study involving 14 children with underweight and 495 children with normal weight and found that children with BMI in the ninth percentile or lower at age 13 months had lower zBMI, and lower HAZ at age 8 years. A prospective cohort study in the US by Black et al^44^ that followed 130 children with weight-for-length *z* score (WLZ) below the fifth percentile and 119 children with normal weight younger than age 25 months and found that children with underweight had lower zBMI and HAZ at age 8 years. In a longitudinal cohort study in the United Kingdom with 11 499 children aged 8 weeks to 9 months with weight gain rate below the fifth percentile, Din et al^45^ found that by age 13 years, children with underweight had lower zBMI and HAZ. Previous studies have been limited by relatively small sample sizes and lack of adjustment for potentially important confounders, such as maternal ethnicity, family income, and breastfeeding duration.^43,44,45^

There is variation in the literature on the definition of underweight in high-income countries. Previous studies have used BMI below the ninth percentile, WAZ below the fifth percentile, WLZ below the fifth percentile, or weight gain rate below the fifth percentile.^25,43,44,45^ Using WAZ or weight SD alone may overestimate undernutrition by identifying children who are thin for their age but not necessarily thin for their height.^43^ The WHO has recommended using the WLZ in the first 2 years of life and zBMI after age 2 years to identify children who are at the highest risk for undernutrition.^5,43,46^ Several studies have found high agreement between WLZ and zBMI in the first 2 years of life, and measurement of BMI from birth through childhood allows for consistent measurement of zBMI growth.^46,47,48,49^ We suggest zBMI less than −2 as a useful definition for future studies.

Strengths of this study include a relatively large, ethnically diverse cohort of healthy children from primary health care practices. Repeated measures of growth from birth through age 10 years allowed for objective measurement of growth at different ages throughout childhood. Additionally, we adjusted for important confounders that were previously identified in the literature.

### Limitations

This study has some limitations. One limitation was using a categorical variable (eg, zBMI less than −2) to define underweight, which does not take into consideration the severity of underweight. For example, some children may have experienced zBMI less than −3, which may have more pronounced effects than zBMI less than −2, although this is rare in high-income countries.^50^ Additionally, although an association was identified up to age 10 years, it was not possible to determine growth during and after puberty and into adulthood.

## Conclusions

This cohort study found that healthy children with underweight in the first 2 years of life had lower zBMI through age 10 years of age. Female sex and a lower growth rate in the first 2 years strengthened these associations while a higher growth rate in the first 2 years attenuated them. Further research is needed to improve recommendations for children with underweight, particularly for girls and those with a lower rate of weight gain in the first 2 years of life.

Future research is needed to explore possible mediators between underweight and growth in later childhood to help understand the mechanisms underlying the observed associations. Ongoing follow-up of growth within this cohort may help understand the outcomes associated with underweight in the first 2 years of life for weight and height through adolescence and into adulthood.

## Notes

Supplement.
The TARGet Kids! Collaboration Members
