# Supplementary material for: Underweight in the First 2 Years of Life and Growth in Later Childhood
Source: JAMA Netw Open. 2022 Jul 29;5(7):e2224417. doi: 10.1001/jamanetworkopen.2022.24417 (PMC9338407; doi:10.1001/jamanetworkopen.2022.24417)
Supplement: Supplement. — The TARGet Kids! Collaboration Members [file jamanetwopen-e2224417-s001.pdf]

Supplemental Online Content: Nonauthor Collaborators
*First name, last name, and suffix (if applicable) are required and will appear in PubMed.

*Group Name(s): TARGet Kids! collaboration							
***First Name and Middle Initial(s)**	***Last Name**	***Suffix (eg, Jr, III)**	Academic Degrees	Institution	Location (city, state/province, country)	Role or Contribution, eg, chair, principal investigator	Group (if more than 1 Group listed in the byline) and/or Subgroup (eg, Steering Committee)
Catherine	Birken		MD			Co-Lead	
Jonathon	Maguire		MD			Co-Lead	
Christopher	Allen		BSc				
Laura	Anderson		PhD				
Dana	Arafeh		MHI				
Mateenah	Jaleel		BSc				
Charles	Keown-Stoneman		PhD				
Natricha	Levy McFarlane		MPhil				
Jessica	Omand		RD, PhD				
Mary	Aglipay		MSc				
Imaan	Bayoumi		MD, MSc				
Cornelia	Borkhoff		PhD				
Sarah	Carsley		PhD				
Katherine	Cost		PhD				
Curtis	D'Hollander		RD, MSc				
Anne	Fuller		MD				
Laura	Kinlin		MD, MPH				
Patricia	Li		MD, MSc				
Pat	Parkin		MD				
Nav	Persaud		MD, MSc				
Izabela	Socynska		RD, MSc				
Shelley	Vanderhout		RD, PhD				
Leigh	Vanderloo		PhD				
Peter	Wong		MD, PhD				
Xuedi	Li		MSc				
Michelle	Mitchell		BA				
Hakimat	Shaibu		MSc				

Supplemental Online Content: Nonauthor Collaborators
*First name, last name, and suffix (if applicable) are required and will appear in PubMed.

***First Name and Middle Initial(s)**	***Last Name**	***Suffix (eg, Jr, III)**	Academic Degrees	Institution	Location (city, state/province, country)	Role or Contribution, eg, chair, principal investigator	Group (if more than 1 Group listed in the byline) and/or Subgroup (eg, Steering Committee)
Yulika	Yoshida-Montezuma		MPH				
Marivic	Bustos		RPN				
Pamela Ruth	Flores		MD				
Martin	Ogwuru		MBBS				
Sharon	Thadani		MLT				
Julia	Thompson		SSRP				
Laurie	Thompson		MLT				
Kardelen	Kurt		BSc				
Ataat	Malick		MD				
Jennifer	Batten						
Jennifer	Chan						
John	Clark						
Maureen	Colford						
Amy	Craig						
Kim	De Castris-Garcia						
Sharon	Dharman						
Anthony	Garcia						
Sarah	Kelleher						
Sandra	Marquez						
Salimah	Nasser						
Tammara	Pabon						
Michelle	Rhodes						
Rafael	Salsa						
Jia	Shin						
Julie	Skelding						
Daniel	Stern						
Kerry	Stewart						
Erika	Sendra Tavares						

Supplemental Online Content: Nonauthor Collaborators
*First name, last name, and suffix (if applicable) are required and will appear in PubMed.

***First Name and Middle Initial(s)**	***Last Name**	***Suffix (eg, Jr, III)**	Academic Degrees	Institution	Location (city, state/province, country)	Role or Contribution, eg, chair, principal investigator	Group (if more than 1 Group listed in the byline) and/or Subgroup (eg, Steering Committee)
Shannon	Weir						
Maria	Zaccaria						
Magdalena	Janus		PhD				
Eric	Duku		PhD				
Caroline	Reid-Westoby		PhD				
Patricia	Raso		MSc				
Amanda	Offord		MSc				
Emy	Abraham		MD				
Sara	Ali		MD				
Kelly	Anderson		MD				
Gordon	Arbess		MD				
Jillian	Baker		MD				
Tony	Barozzino		MD				
Sylvie	Bergeron		MD				
Gary	Bloch		MD				
Joey	Bonifacio		MD				
Ashna	Bowry		MD				
Caroline	Calpin		MD				
Douglas	Campbell		MD				
Sohail	Cheema		MD				
Elaine	Cheng		MD				
Brian	Chisamore		MD				
Evelyn	Constantin		MD				
Karoon	Danayan		MD				
Paul	Das		MD				
Viveka	De Guerra		MD				
Mary Beth	Derocher		MD				
Anh	Do		MD				
Kathleen	Doukas		MD				
Anne	Egger		BScN				
Allison	Farber		MD				

Supplemental Online Content: Nonauthor Collaborators
*First name, last name, and suffix (if applicable) are required and will appear in PubMed.

***First Name and Middle Initial(s)**	***Last Name**	***Suffix (eg, Jr, III)**	Academic Degrees	Institution	Location (city, state/province, country)	Role or Contribution, eg, chair, principal investigator	Group (if more than 1 Group listed in the byline) and/or Subgroup (eg, Steering Committee)
Amy	Freedman		MD				
Sloane	Freeman		MD				
Sharon	Gazeley		MD				
Karen	Grewal		MD				
Charlie	Guiang		MD				
Dan	Ha		MD				
Curtis	Handford		MD				
Laura	Hanson		BScN, RN				
Leah	Harrington		MD				
Sheila	Jacobson		MD				
Lukasz	Jagiello		MD				
Gwen	Jansz		MD				
Paul	Kadar		MD				
Lukas	Keiswetter		MD				
Tara	Kiran		MD				
Holly	Knowles		MD				
Bruce	Kwok		MD				
Piya	Lahiry		MD				
Sheila	Lakhoo		MD				
Margarita	Lam-Antoniades		MD				
Eddy	Lau		MD				
Denis	Leduc		MD				
Fok-Han	Leung		MD				
Alan	Li		MD				
Patricia	Li		MD				
Roy	Male		MD				
Aleks	Meret		MD				
Elise	Mok		MD				
Rosemary	Moodie		MD				
Katherine	Nash		BScN, RN				

Supplemental Online Content: Nonauthor Collaborators
*First name, last name, and suffix (if applicable) are required and will appear in PubMed.

*First Name and Middle Initial(s)	*Last Name	*Suffix (eg, Jr, III)	Academic Degrees	Institution	Location (city, state/province, country)	Role or Contribution, eg, chair, principal investigator	Group (if more than 1 Group listed in the byline) and/or Subgroup (eg, Steering Committee)
James	Owen		MD				
Michael	Peer		MD				
Marty	Perlmutar		MD				
Navindra	Persaud		MD				
Andrew	Pinto		MD				
Michelle	Porepa		MD				
Vikky	Qi		MD				
Noor	Ramji		MD				
Danyaal	Raza		MD				
Katherine	Rouleau		MD				
Caroline	Ruderman		MD				
Janet	Saunderson		MD				
Vanna	Schiralli		MD				
Michael	Sgro		MD				
Hafiz	Shuja		MD				
Farah	Siam		MD				
Susan	Shepherd		MD				
Cinntha	Srikanthan		MD				
Carolyn	Taylor		MD				
Stephen	Treherne		MD				
Suzanne	Turner		MD				
Fatima	Uddin		MD				
Meta	van den Heuvel		MD				
Thea	Weisdorf		MD				
Peter	Wong		MD				
John	Yaremko		MD				
Ethel	Ying		MD				
Elizabeth	Young		MD				
Michael	Zajdman		MD				
Peter	Juni		MD				

Supplemental Online Content: Nonauthor Collaborators
*First name, last name, and suffix (if applicable) are required and will appear in PubMed.

***First Name and Middle Initial(s)**	***Last Name**	***Suffix (eg, Jr, III)**	Academic Degrees	Institution	Location (city, state/province, country)	Role or Contribution, eg, chair, principal investigator	Group (if more than 1 Group listed in the byline) and/or Subgroup (eg, Steering Committee)
Gurpreet	Lakhanpal		MSc				
Gerald	Lebovic		PhD				
Audrey	Stitt		MSc				
Kevin	Thorpe		MMath				
Ifeayinchukwu (Shawn)	Nnorom		BSc				
Esmot ara	Begum		PhD				
Rita	Kandel		MD				
Michelle	Rodrigues		PhD				
Andrea	Djolovic						
Raya	Assan						
Homa	Bondar						
